# Efficient Assessment of Tumor Vascular Shutdown by Photodynamic Therapy on Orthotopic Pancreatic Cancer Using High-Speed Wide-Field Waterproof Galvanometer Scanner Photoacoustic Microscopy

**DOI:** 10.3390/ijms25063457

**Published:** 2024-03-19

**Authors:** Jaeyul Lee, Sangyeob Han, Til Bahadur Thapa Magar, Pallavi Gurung, Junsoo Lee, Daewoon Seong, Sungjo Park, Yong-Wan Kim, Mansik Jeon, Jeehyun Kim

**Affiliations:** 1 School of Electronic and Electrical Engineering, College of IT Engineering, Kyungpook National University, Daegu 41566, Republic of Korea; jlee350@mgh.harvard.edu (J.L.); syhan850224@knu.ac.kr (S.H.); junsoo.lee@uci.edu (J.L.); smc7095@knu.ac.kr (D.S.); jeehk@knu.ac.kr (J.K.); 2 Organic Nanoelectronics Laboratory, KNU Institute for Nanophotonics Applications (KINPA), Department of Chemical Engineering, School of Applied Chemical Engineering, Kyungpook National University, Daegu 41566, Republic of Korea; 3 Institute of Biomedical Engineering Research, Kyungpook National University, Daegu 41566, Republic of Korea; 4 Dongsung Cancer Center, Dongsung Bio Pharmaceutical Co., Ltd., Daegu 41061, Republic of Korea; til09@ds-pharm.co.kr (T.B.T.M.); gp20@ds-pharm.co.kr (P.G.); 5 Laser Application Center, Institute of Advanced Convergence Technology, Kyungpook National University, Daegu 41061, Republic of Korea; sungjo@iact.or.kr

**Keywords:** photoacoustic microscopy, waterproof galvanometer scanner, photodynamic therapy, pancreatic cancer, chlorin e6

## Abstract

To identify the vascular alteration by photodynamic therapy (PDT), the utilization of high-resolution, high-speed, and wide-field photoacoustic microscopy (PAM) has gained enormous interest. The rapid changes in vasculature during PDT treatment and monitoring of tumor tissue activation in the orthotopic pancreatic cancer model have received limited attention in previous studies. Here, a fully two-axes waterproof galvanometer scanner-based photoacoustic microscopy (WGS-PAM) system was developed for in vivo monitoring of dynamic variations in micro blood vessels due to PDT in an orthotopic pancreatic cancer mouse model. The photosensitizer (PS), Chlorin e6 (Ce6), was utilized to activate antitumor reactions in response to the irradiation of a 660 nm light source. Microvasculatures of angiogenesis tissue were visualized on a 40 mm^2^ area using the WGS-PAM system at 30 min intervals for 3 h after the PDT treatment. The decline in vascular intensity was observed at 24.5% along with a 32.4% reduction of the vascular density at 3 h post-PDT by the analysis of PAM images. The anti-vascularization effect was also identified with fluorescent imaging. Moreover, Ce6-PDT increased apoptotic and necrotic markers while decreasing vascular endothelial growth factor (VEGF) expression in MIA PaCa-2 and BxPC-3 pancreatic cancer cell lines. The approach of the WGS-PAM system shows the potential to investigate PDT effects on the mechanism of angiographic dynamics with high-resolution wide-field imaging modalities.

## 1. Introduction

Pancreatic cancer is a well-known and challenging disease to be diagnosed at an early stage and is the seventh leading cause of cancer-related deaths worldwide. Due to the difficulty of surgical treatments, the relative survival rate after five years of diagnosis of pancreatic ductal adenocarcinoma is less than 5%, while that in the case of surgically resected patients is about 17% [1,2,3]. Although the recurrence rate is high, approximately 20% of patients can achieve a five-year survival following a successful surgical removal of the tumor [4,5,6]. However, the presence of other internal organs, such as the stomach, duodenum, and spleen, in proximity to the pancreas, barricades the straightforward surgical procedure for pancreatic cancer treatments [7,8,9]. Radiation treatment and chemotherapy have been frequently utilized to overcome challenging surgical procedures [10]. Radiation is generally used to alleviate symptoms and prolong life [11,12]. However, radiation affects not only tumor cells but also surrounding normal tissues, leading to side effects such as disorders of the immune system [13,14]. Chemotherapy inhibits the proliferation of cancer cells by introducing carcinostatic substances within a specific timeframe, but still also has some adverse effects [15,16,17]. Anticancer drugs, such as 5-fluorouracil and gemcitabine, are options that are generally not yet deemed effective in treating pancreatic cancer [18,19].

Photodynamic therapy (PDT) has emerged as one of the most promising treatment options for pancreatic cancers [20,21,22,23,24]. The treatment approach of PDT relies on the generation of reactive oxygen species (ROS) and their subsequent toxic effects after the excitation of photosensitizer (PS) by using light of a specific wavelength in the targeted region [25,26]. PDT leads to cancer cell death by necrosis and/or apoptosis via interactions between ROS and biomolecules [27,28]. The PDT-induced damage to endothelial cells triggers a cascade of events, including platelet aggregation and the activation of clotting factors, leading to the formation of blood clots within the treated vessels. This process, known as thrombosis, contributes to the shutdown or occlusion of the targeted blood vessel [29]. However, studies concerning vascular dysfunction resulting from Ce6-mediated-PDT, including changes in micro blood vessels within a broad area of orthotopic pancreatic tumor tissues, have been limited [30,31]. Fingar, for the first time in 1996, described the PDT effects in vascular stasis and blood flow stasis [32]. Nevertheless, it has been challenging to identify microvascular changes brought on by PDT treatment using traditional imaging methods in anti-tumor research [25,33].

Computed tomography (CT), magnetic resonance imaging (MRI), and positron emission tomography (PET) have been used to evaluate the antitumor effects of PDT [34,35,36,37]. However, these time-consuming techniques suffer from limited spatial resolution during in vivo PDT assessments. Optical Doppler tomography (ODT) can be used to visualize the size and flow of blood vessels [38,39]. However, ODT is limited by its low penetration depth due to strong light scattering and absorption [40]. Therefore, to assess the vascular effects of PDT, high-resolution and high-contrast imaging methods are beneficial in understanding the mechanisms of pancreatic cancer therapy [41,42].

Photoacoustic microscopy (PAM) is a promising optical imaging technique for vascular imaging [43,44,45,46,47]. PAM relies on optical absorption for imaging, enabling high-resolution visualization without the assistance of contrast agents [48,49,50]. Pulsed lasers induce transient thermal expansion on the target tissue leading to the generation of acoustic waves which are detected by an ultrasonic transducer and processed for the visualization of blood vascular networks and functional analysis of biological tissues [51,52]. High-speed wide-field photoacoustic imaging has been extensively utilized for the study of tumor microenvironments [53,54,55,56]. Utilization of PAM together with PDT in cancer research has been proven advantageous by addressing issues related to time consumption, resolution, and contrast as compared to conventional imaging techniques mentioned earlier [55,56]. Nevertheless, studies on an orthotopic pancreatic cancer model utilizing PAM for wide-area and high-resolution modalities are scarce [54,57].

Herein, we report an assessment of vascular disruption caused by Chlorin e6 (Ce6)-mediated PDT in an orthotopic pancreatic cancer model using a high-speed wide-field waterproof galvanometer scanner-based photoacoustic microscopy (WGS-PAM) system. A well-known PS, Ce6 [58,59,60] has been used together with the light of a 660 nm wavelength having a power of 200 mW/cm^2^. Ce6, a second-generation photosensitizer, has been extensively studied for treating different types of cancer, such as pancreatic, skin, colon, and ovarian cancer [23,61,62]. However, very little research about the underlying vascular mechanism of Ce6-mediated PDT encouraged us to study its vascular shutdown efficacy utilizing the WGS-PAM system. The diversification of micro blood vessels was dynamically observed using high-resolution imaging with WGS-PAM, including transient hemorrhaging in the case of a leaky vasculature. The measured maximum amplitude projection (MAP) of the WGS-PAM images was qualitatively and quantitatively assessed for vascular shutdown effects during the 3 h of monitoring after PDT. The microvasculature was damaged by the induced thrombus and the ROS impact of vascular-targeted PDT treatment. The effects assessed using epifluorescence and luminescence suggested that a sufficient amount of PS was present in the pancreatic tumor region for 3 h. To the best of our knowledge, this work elucidates the impact of Ce6-associated PDT on the dynamics of blood flow during pancreatic cancer treatment. Additionally, it highlights the valuable application of PAM techniques in achieving detailed, high-contrast imaging of vascularization across a wide area of tumor tissue. This can give us a better comprehension of the complicated mechanisms that are involved during Ce6-PDT-mediated tumor vascular destruction.

## 2. Results

### 2.1. Western Blot Analysis for the Pancreatic Tumor Cells

**Figure 1:**
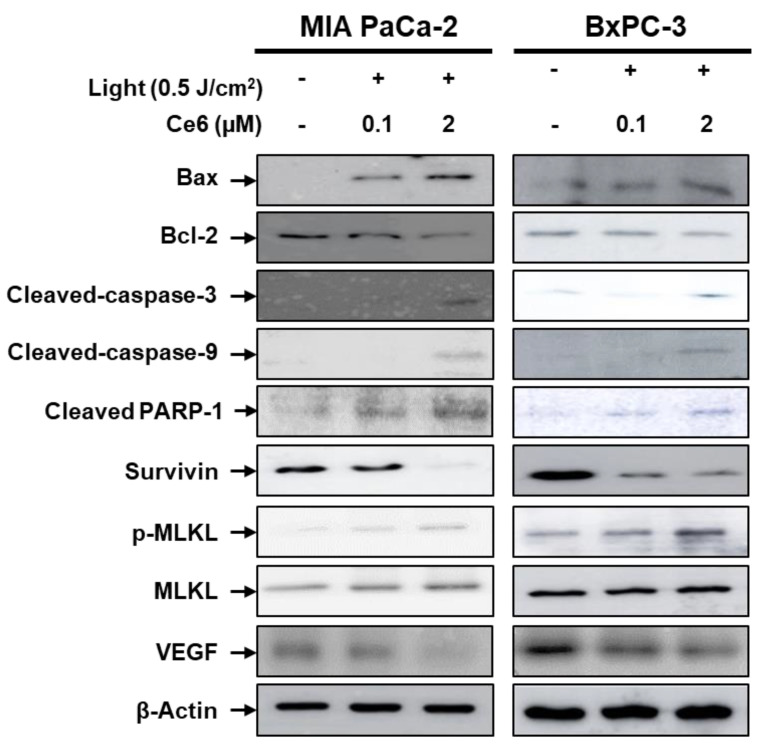
Western blot analysis of apoptosis and necroptosis-related proteins for pancreatic cancer cell lines at 24 h post-PDT. The protein levels of Bax, Bcl-2, cleaved caspase-3, cleaved caspase-7, cleaved PARP-1, survivin, p-MLKL, MLKL and VEGF protein levels in MIA PaCa-2 and BxPC-3-luc cells were determined by Western blotting. β-Actin was used as an internal control.

To evaluate the Ce6-PDT effects on apoptosis, necroptosis-related and VEGF protein expression in vitro, two pancreatic cancer cell lines were included in our study: MIA PaCa-2 cells and BxPC-3-luc cells, as shown in Figure 1. The MIA PaCa-2 cells are compared as a general reference to pancreatic cancer cells with Bx-PC-3-luc cells. Our results show that Ce6-PDT-induced cell death in pancreatic cells was marked by a significant downregulation of the antiapoptotic proteins (Bcl-2) and by a significant upregulation of proapoptotic marker proteins like Bax, cleaved caspase-9, and cleaved caspase-3 in a concentration-dependent manner. Furthermore, cell treatment with Ce6-PDT also dose-dependently inhibited cleaved PARP-1 and surviving protein expressions compared with the untreated groups. The primary mechanism of cell death for PDT is apoptosis, but necrotic pathways can also regulate cell death [63]. Therefore, the role of necroptosis in Ce6-PDT-induced cell death was explored with the elevated phosphorylation of MLKL. Similar outcomes were obtained in mouse melanoma tumors treated with Ce6-PDT (Figure S5). The qualitative analysis of the Western blot illustrates that Ce6-PDT was able to activate apoptosis and necroptosis pathways in pancreatic cancer cells. Additionally, Ce6-PDT treatment also interfered with angiogenic processes, as a reduction of the VEGF level was found in a concentration-dependent manner in both pancreatic cancer cell lines. In our study, we employed a greater dosage of Ce6-PDT in our investigation, and it is well established that these higher PDT dosages lead to more significant damage to the areas where VEGF protein is biosynthesized, namely the Golgi bodies and the endoplasmic reticulum [64,65,66]. Ce6 must have concentrated in these regions and may have harmed the production of VEGF [67]. Therefore, Ce6-PDT confirmed that it not only induces apoptosis in malignant cells but also affects tumor vasculature.

### 2.2. Circulation of Photosensitizer

**Figure 2:**
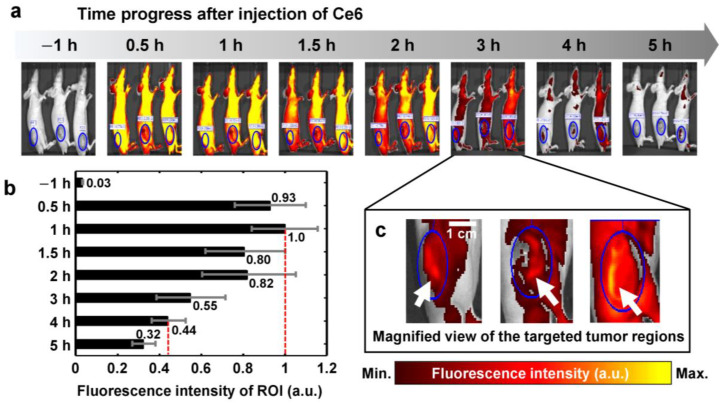
Ce6 circulation monitoring after injection. (**a**) Fluorescence images of orthotopic pancreatic cancer model using Ce6 for 5 h. (**b**) Normalized fluorescence intensity at the region of interest, which is indicated by the blue-color circles of (**a**). (**c**) Magnified images of targeted tumor tissues.

After learning about the Ce6-PDT effects in cell death molecules and VEGF in pancreatic cancer cells, we ascertained the Ce6-PDT effects in the orthotopic mice model of pancreatic cancer. Orthotopic mice models of pancreatic cancer were established by injecting the BXPC-3-luc cell line. Tumor growth was monitored by the in-vivo imaging (Figure S7). Circulation of the photosensitizer was determined by fluorescence imaging performed for five consecutive hours after injecting Ce6 into the mice as depicted in Figure 2. The progressive distribution of Ce6 is shown in Figure 2a and the region of interest is indicated at each corresponding time interval. The ROIs (blue circles of Figure 2a) were manually classified. The area of ROIs was 1.5–3.5 ${cm}^{2}$ including tumor tissues and the around regions. The average fluorescence intensity within the region enclosed by blue oval windows is numerically assessed and plotted in Figure 2b. The maximum normalized intensity as indicated by red-dashed line, was identified 1 h post Ce6 injection. Subsequently, 80% and 82% of normalized maximum intensity were observed for a successive 2 h. The intensity was reduced to less than 50%, 4 h after the injection (red-dash line). The white color arrows indicated a distinctly brighter area of the tumor region in Figure 2c. The result suggests that the sufficient amount of Ce6 was present at the tumor site for successful PDT treatment after 1 h.

### 2.3. Angiogenic Sham Images of Tumor Tissues

**Figure 3:**
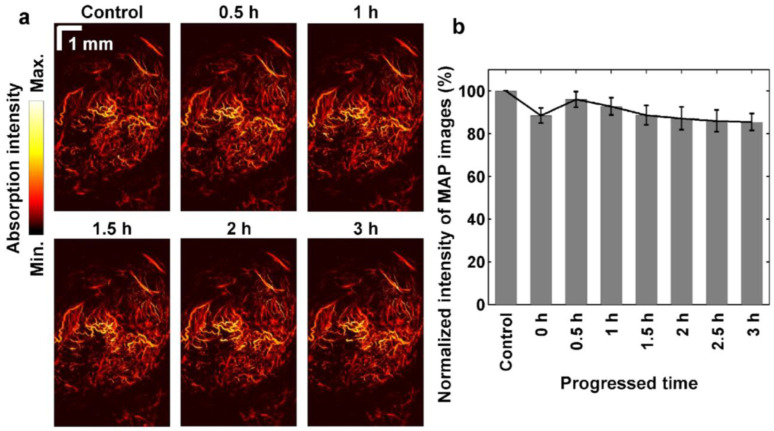
Maximum amplitude projection images of photoacoustic microscopy in shams without photodynamic therapy. (**a**) Representative PAM-MAP images of sham, (**b**) normalized averaged intensity of sham MAP images, which includes only anesthesia, only injection and only illumination.

Three sham groups for the anesthesia, injection of photosensitizer, and illumination of activating light cases were assessed using the WGS-PAM system without the PDT activation to compare the PDT effects. The anesthesia group was conducted only for anesthesia and dissection for PAM imaging. The photosensitizer injection group was monitored using the PAM system after the circulation of Ce6 without the activating irradiation. The light illumination group was visualized using the PAM system without any injection of photosensitizer. Figure 3 shows representative MAP images of the sham for 3 h. The extensive microvasculature of neovascularization was visualized in angiogenetic tumor tissues. The formation of abundant microvessels on the orthotopic pancreas tissues can be confirmed through the acquired high-resolution images in Figure 3a. The intensity variation of PAM images was observed especially at 0 h in Figure 3b. The shams (only injection of photosensitizer; only irradiation) were visualized at 0 h after the transient intervention (movements from the injection and irradiation) of each of the only-injection and only-irradiation movements. The noise frames from the transient movement were manually cropped before the intensity normalization. The average (±standard deviation) absorption intensity of MAP images of shams (the anesthesia, injection of photosensitizer, and illumination shams) shows 100 (±0)% (control), 88.5 (±3.5)%, 96.1 (±3.7)%, 92.8 (±4.1)%, 88.7 (±4.6)%, 87.2 (±5.4)%, 86.0 (±5.1)%, and 85.5 (±4.0)% at 30 min intervals for 3 h. Overall absorption intensity was decreased due to prolonged anesthesia with the dissected skin. The respiration of shams was affected by each condition because the ratio of oxyhemoglobin and deoxyhemoglobin has a different absorption coefficient to the imaging light [68,69,70]. Finally, the average intensity of PAM images of shams decreased to 85.5% ± 4.0% after 3 h.

### 2.4. Capturing Hemorrhage Phenomena Caused by PDT

**Figure 4:**
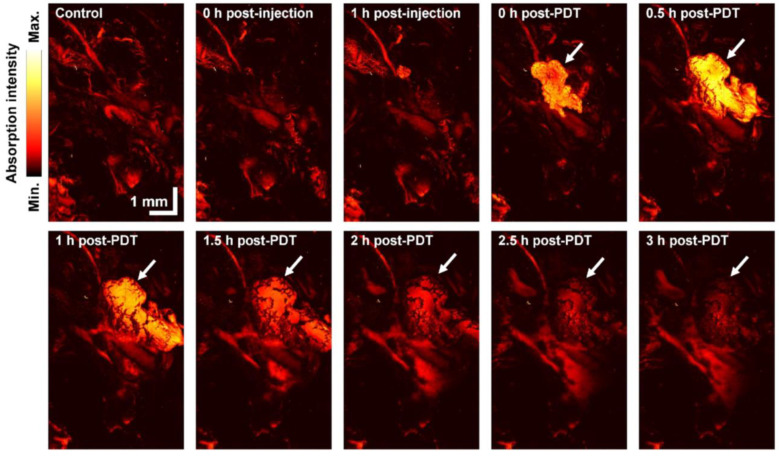
Maximum amplitude projection images of photoacoustic microscopy with hemorrhaging tumor tissues caused by the PDT. The white arrow indicates the hemorrhage point.

The dynamic variation of post-PDT hemorrhage was observed through the WGS-PAM system in Figure 4. The MAP images of control and post-injection subjects showed no significant alterations of structural blood vessels and absorption intensity. Both the control and post-injection images indicate particular branching of microvasculature from relatively thick vessels. Hemorrhaging was observed at 0 h post-PDT as indicated by a white arrow. The absorption intensity reached a maximum, at 0.5 h post-PDT. The central part of the blood was brighter than the sides. After 1 h, the maximum blood volume was observed, but the intensity was decreased as compared to 0.5 h post-PDT. The overall intensity and the number of blood vessels also gradually decreased in the surrounding area during the 3 h of the monitoring period. It shows the transient moment of the hemorrhage and the discontinuation of the hemorrhage. Previous findings suggest that PDT causes thrombosis, which leads to micro blood vessel constriction [71,72,73]. Hence the microvasculature was clogged due to the PDT effect. The hemorrhage was observed only once among five times PDT treatment cases. The blood exudating was presumably not desired. However, the WGS-PAM system showed the capability to visualize the phenomena.

### 2.5. Assessments of PAM Images by the Progress of PDT

**Figure 5:**
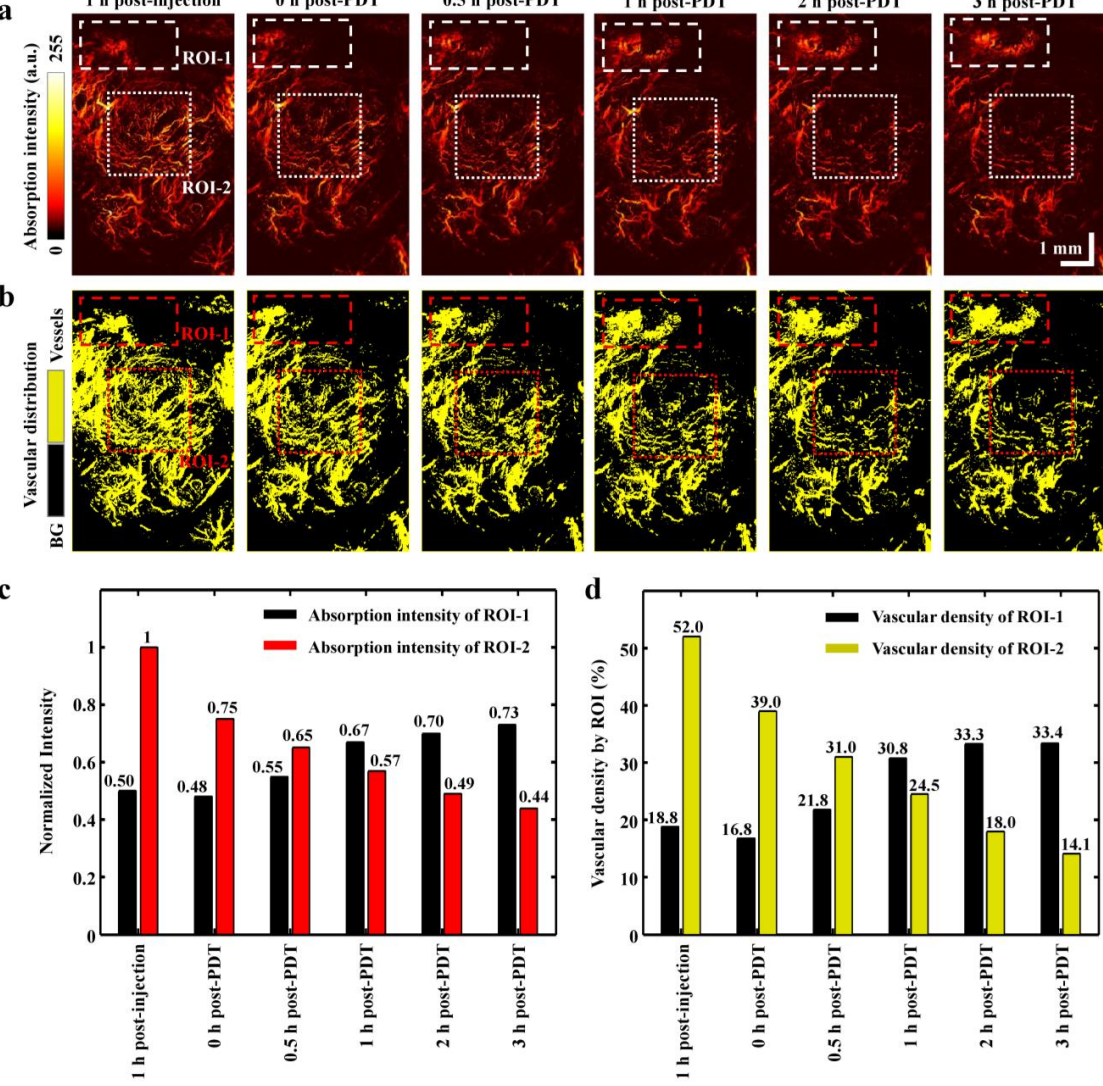
PDT analysis based on photoacoustic microscopy images. (**a**) Maximum amplitude projection images of photoacoustic microscopy of the monitored image after PDT. (**b**) Vasculature classification images through an intensity-based image processing. (**c**) Comparison of the absorption intensity analysis with ROI-1 and ROI-2. (**d**) Comparison of the vascular distribution density by each ROI of MAP images. ROI-1 indicates relatively big vessels. ROI-2 indicates microvessels.

The PAM images represent the variation of absorption intensity and vascular distribution density due to the vascular destructive effect of PDT as shown in Figure 5. The MAP images indicate the vascular regions having absorption intensity on the pancreas tumor tissue (Figure 5a). Then, the vascular regions are converted to yellow-colored vessels to clearly identify the vascular distribution density through intensity-based image processing as shown in Figure 5b. The dashed rectangular regions indicate the region of interest one (ROI-1; big vessels), and the dotted square designates the region of interest two (ROI-2; microvessels). ROI-1 indicates the relatively large vessels, which are compared to microvessels of ROI-2. The normalized absorption intensity of the big vessel area shows 0.73 at 3 h post-PDT as shown in Figure 5c. The intensity of the microvessel area indicates a continuous reduction to 0.44 for 3 h. The absorption intensity of the microvessels of ROI-2 decreased by 56% between the 1 h post-injection and the 3 h post-PDT. On the contrary, the absorption intensity of ROI-1 was increased by 46% in the same time interval.

The vascular densities of ROI-1 and ROI-2 are described in Figure 5d. The vascular density of ROI-1 increased from 18.8% to 33.4% between the 1 h post-injection and 3 h post-PDT. Meanwhile, the vascular density of the ROI-2 microvessels was found to be decreased by 37.9%. The variation tendency between the absorption intensity and vascular density was identified to be equal (Figure 5c,d).

These parameters (absorption intensity and vascular density) were not changed to the controls, as we checked in the shams. For the representative visualization, the 1 h post-injection indicates the best positioning after the PDT treatment for the PAM imaging. Furthermore, the PAM imaging of the pre-PDT shows the efficient comparison between the PDT effect on microvessels in the smallest time interval of the procedure.

**Figure 6:**
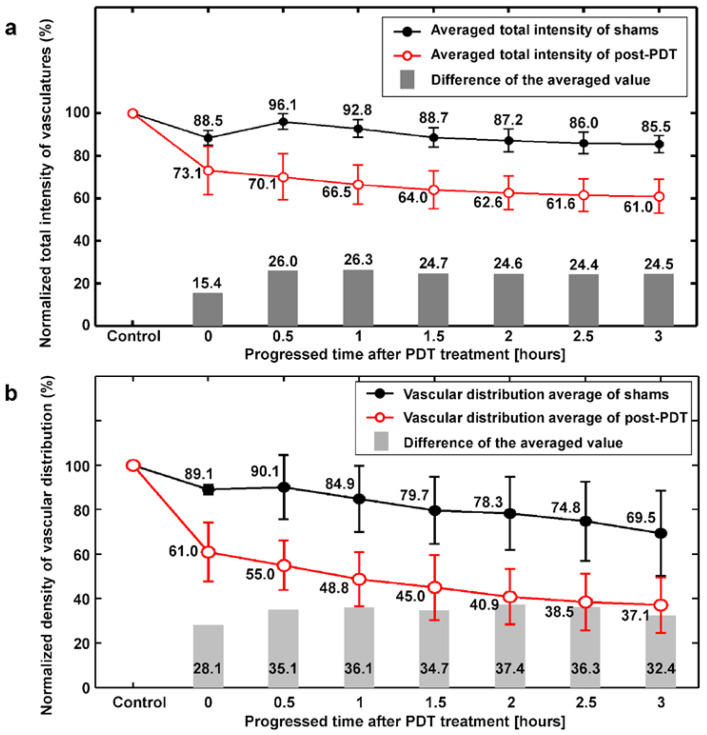
Comparison of averaged intensity and vascular density of PAM images for all cases at different time intervals after PDT. (**a**) Normalized total intensity and the difference between shams and post-PDT for 3 h. (**b**) Normalized density of vascular distribution and the difference between shams and post-PDT for 3 h.

The total averaged analysis of three shams and four PDT cases are quantitatively indicated by the normalized intensity and normalized density of vasculatures (Figure 6). The whole area (5 mm × 8 mm) of PAM images was analyzed without ROI segmentations at each imaging point (30 min intervals). The intensity of vasculature illustrates the hemoglobin concentration of blood of PAM signals. The reduction of vascular intensity shows the gradual blood signal weakness. The vascular density indicates the ratio of the vascular distribution area (yellow color) divided by the field of view of the PAM image such as Figure 5b. The gradual reduction of vascular density represents the close-down of vessels and the decrease in inner diameter of vascular lumens along the time intervals. The average intensities of both the shams and post-PDT cases were decreased in a time-dependent manner, as shown in Figure 6a. The 14.5% reduction of sham intensity was observed after 3 h of monitoring. It seems that the dissection procedure of shams affects the intensity variation regardless of PDT illumination. The PDT cases represent a relatively clearer reduction as compared to the shams. The result shows the vascular intensity reduction of 39.0% at 3 h post-PDT. The intensity difference of shams and 3 h post-PDT was 24.5%. The averaged vascular distribution between the shams and the post-PDT is compared in Figure 6b. The vascular density of sham PAM images decreased to 69.5% after 3 h monitoring. While at the same time interval, the 3 h post-PDT case shows 37.1%. The difference of averaged vascular density was 32.4% for 3 h. The variations in intensity and vascular density indicate the rapid change caused by the PDT at the initial 30 min as compared to the variation after 1 h. The correlation between the intensity and the vascular density difference of the shams and post-PDT was also plotted (Figure S4).

The WGS-PAM system presented a high-resolution wide-field modality in the in vivo vascular images of orthotopic tumor tissue. The variation of vascular intensity and density qualitatively confirmed that the microvessels were primarily blocked and then the relatively thick vessels were enlarged. Averaged analysis showed that the intensity decreased by 15.4% and 24.5% and the density decreased by 28.1% and 32.4% at 0 h and 3 h post-PDT, respectively. The results obtained with high-resolution wide-field PAM images suggested that the PDT induced vascular damage on the tumor tissue region. Then, the one-tailed paired *t*-test was utilized as a statistical evaluation to compare shams and post-PDT. Each *p*-value of averaged intensity and vascular distribution showed <0.001, which is a statistically meaningful value, between shams and post-PDT.

### 2.6. Fluorescence Imaging for Comparison with PAM Images

**Figure 7:**
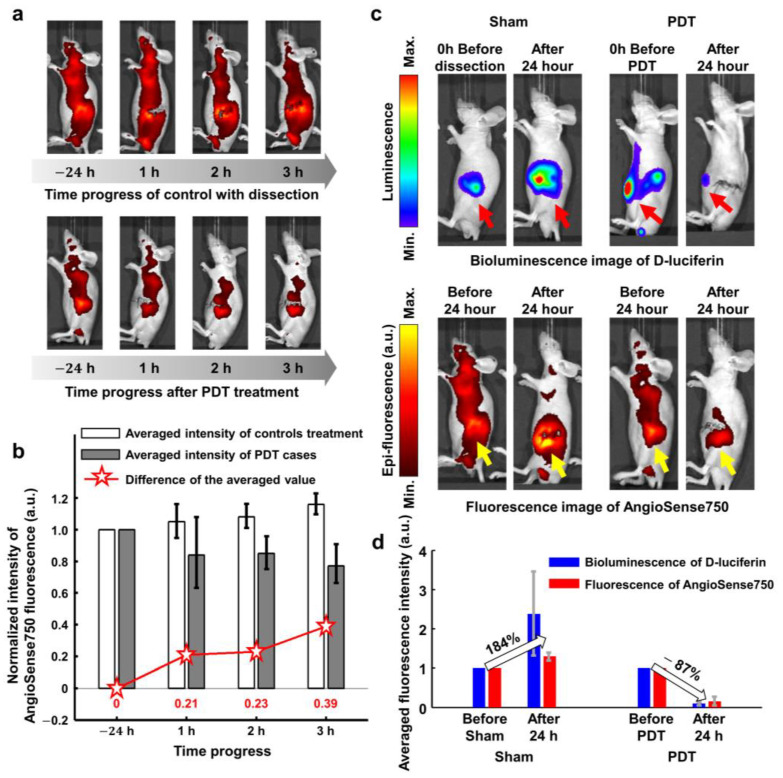
Assessment of PDT efficacy compared with shams. (**a**) Fluorescence images of AngioSense750 compared with sham and PDT. (**b**) Normalized intensity comparison of AngioSense750 up to 3 h after PDT. (**c**) Bioluminescence images and fluorescence images by the respective D-luciferin and AngioSense750 for one day after PDT. (**d**) Averaged intensity comparison of sham and PDT cases one day after PDT treatment.

Sham and PDT cases were compared by epi-fluorescence and bioluminescence images, as shown in Figure 7. The fluorescence intensity of the control gradually increased after 3 h, and the contrast of the tumor region increased simultaneously as well. PDT cases showed a decrease in fluorescence intensity (Figure 7a). The intensity difference of 39% was observed between shams and 3 h post-PDT (Figure 7b). Then, the *p*-value of the one-tailed paired *t*-test between the shams and PDT showed <0.05 value in the averaged fluorescence intensity. Additionally, the PDT effects were assessed using bioluminescence images of D-luciferin, as shown in Figure 7c,d. The intensity of the tumor region of shams was increased as compared to the PDT cases after one day of treatment (Figure 7c). The red and yellow arrows indicate the tumor region in the bioluminescence and fluorescence images, respectively. An increase in intensity by 184% in the dissection-only case of the shams and a decrease in 87% in the PDT treatment case were observed (Figure 7d).

The two types of imaging, Angiosense750 and D-luciferin were utilized to verify the PDT effect with relatively short-term monitoring. The results of fluorescence imaging also displayed the decrease in intensity and region of signals by PDT, which corroborates to PAM results. 

### 2.7. Histological Validation for the Pancreatic Tumor Tissue

**Figure 8:**
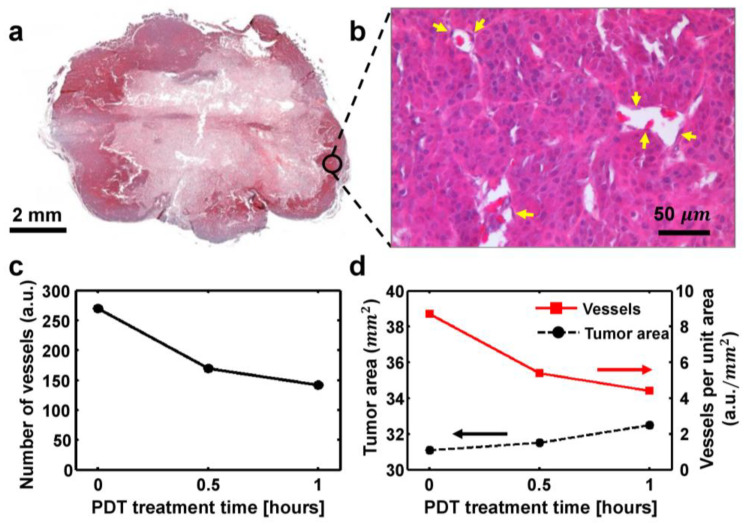
H&E stained tumor area and vessel analysis of the orthotopic pancreas cancer. (**a**) Picture of the sectioned tumor tissue region, (**b**) H&E stained microscopy image, (**c**) counted numbers of capsule and tumor vessels, (**d**) tumor area and vessels per unit area analyzed for 1 h post-PDT. The yellow arrows indicate the microvessels.

The vascular shutdown effect of the PDT treatment was validated by the histological analysis as shown in Figure 8. The dissected tumor tissue was pictured, and the hematoxylin–eosin (H&E) staining was conducted for visualization (Figure 8a). The H&E stained microscopic image shows the microvessels indicated by the yellow arrows (Figure 8b). The counted numbers of capsule and tumor vessels elucidate the vascular reduction after PDT as shown in Figure 8c. The microvessel and capsules counting in the tumor tissue area were performed by a pathologist. The numbers of microvessels in the tumor region were found to be 270, 170, and 142 after 0 h, 0.5 h, and 1 h PDT, respectively. The tumor area was calculated as 31.1 mm^2^, 31.5 mm^2^, 32.5 mm^2^, at 0 h, 0.5 h, and 1 h post-PDT. For the same time intervals, the counted vessels per unit area decreased to 8.7 vessels/mm^2^, 5.4 vessels/mm^2^, and 4.4 vessels/mm^2^ after the PDT treatment as shown in Figure 8d. The microvessel reduction and tumor area expansion were observed after an initial 1 h of PDT.

## 3. Discussion

Targeting the tumor vasculature presents a compelling therapeutic approach as these vessels provide oxygen and nutrients crucial for tumor cell survival and serve as primary pathways for tumor metastasis [74]. The mechanism of PDT-induced hemostasis has been extensively investigated by various researchers. In vascular PDT, the primary target seems to be the endothelial cell [75,76,77]. Upon injection, the PS becomes partially internalized into the endothelial cells. The irradiation of light then causes ROS-mediated damage to the cytoskeleton and other cellular structures. Consequently, PDT triggers the rounding and contraction of endothelial cells, leading to the rupture of interendothelial cell tight junctions and exposing the subendothelial basement membrane. PDT prompts the release of the von Willebrand factor [78], thromboxane, prostacyclin, and other factors, including the vascular endothelial growth factor (VEGF), resulting in elevated vascular permeability, exudation, clotting, and vasoconstriction [75,76,79]. Activated platelets adhere to the damaged vessel wall at the gaps formed between rounded endothelial cells. Platelets aggregate, forming a plug in the vessel, which ultimately culminates in the development of a fibrin-stabilized thrombus and vasoconstriction [80]. The ultimate outcome is hemostasis with tissue hypoxia. It was observed that PDT can more easily close smaller vessels than larger vessels, as seen in the human choroid [29]. Generally, the occlusion of a vessel depends on the quantity and location of the photosensitizer and the administered light. Larger-diameter vessels, where stasis has been noted, may reopen, allowing for perfusion re-establishment. Antivascular therapy employed as vascular-disrupting agents (VDAs) aims to destroy established tumor vasculature, leading to the death of secondary tumor cells and a rapid, extensive reduction in tumor blood flow [81]. One such VDA under clinical trial investigation is combretastatin A4 3ʹ-O-phosphate (CA4P) [82]. CA4P has the capacity to induce interphase microtubule depolymerization, actinomyosin contractility, and reorganization of the actin cytoskeleton through activation of the RhoA/Rho-associated protein kinase (ROCK) pathway [83].

Research indicates that employing Ce6 in PDT facilitates deeper tissue necrosis due to its absorption wavelength falling within the range from 650 to 950 nm. Studies have revealed that Ce6’s mode of action relies on its accumulation in the vasculature rather than the tumor tissue itself. The primary explanation for its effectiveness lies in the disruption of the vascular system of tumors through PDT. Ce6 exhibits a strong absorption range from 640 to 680 nm, peaking around 660 nm, and emits intense fluorescence within the 640 to 700 nm spectrum. Despite the extensive clinical use of the first-generation photosensitizer, porfimer sodium (Photofrin^®^), it induces prolonged skin photosensitivity, and its limited tissue penetration is due to its short absorption wavelength [84,85]. On the other hand, chlorin e6, a second-generation photosensitizer, is rapidly cleared from the body, requiring a shorter sunshade period (2 weeks) in comparison to first-generation photosensitizers (4 weeks) [86]. Therefore, we have attempted to study the vascular shutdown efficacy of Ce6-PDT.

Pancreatic cancer is one of the most lethal cancers, and currently, surgical resection combined with vascular resection remains the sole viable treatment option for pancreatic cancer. Under such circumstances, surgical resection and the WGS-PAM system can be used in conjunction with Ce6-PDT treatment to treat aggressive pancreatic tumors [87]. In our study, we inoculated human pancreatic cancer in athymic mice to determine the effects of Ce6-PDT. Nude mice have a limited population of leftover T cells that can cause observable immunological responses. According to the previous evidences, B cell, natural killer cell, and macrophage counts are comparable to, or greater than, those of the same strain of euthymic mice even though they are in an athymic state. In addition, athymic nude mice have residual immunity against human tumor xenografts [88]. The WGS-PAM system was not used to conduct the long-term monitoring of the orthotopic pancreatic cancer models because of the contamination of tumor tissue by acoustic gel in this experiment. Nevertheless, this study was focused on the evaluation of the prompt interval efficacy on orthotopic pancreatic cancer tissue using the WGS-PAM system. This system enables high-speed wide-field imaging and allows for 3 h of in vivo monitoring following PDT through intensity-based analysis.

The findings suggest that Ce6 is a vascular active agent that, upon light, causes a fast vascular shutdown. Imaging modalities like PA provide deeper insights into therapeutic mechanisms of action including the monitoring of vessel size, as shown by Shao et al. [41]. Utilizing such a PA imaging system in the pancreatic tumor model, we studied the impact of the Ce6-mediated PDT effect on the tumor vasculature. The effects of Ce6-PDT began immediately following treatment. At 0.5 h PDT, there was maximum intensity in functional vasculature, which continued to decline 0.5 h later; 3 h later, intensity and functional blood vessels were all decreased in the tumor. More precisely, blood flow stasis causes a decrease in the amount of oxygen carried by the blood, which causes tumor regression. They attributed this increase to the narrowing of the lumen following vasoconstriction [89]. Our study also found that though vascular damage was observed immediately after PDT exposure, cell death was detected only after 1 h of PDT exposure in vitro (Figure S6). Previous research has demonstrated that in the vascular bed of malignancies, endothelial cell apoptosis occurs prior to tumor cell apoptosis after cytotoxic treatment [90]. Our results are consistent with those of Lee et al. (2015), who discovered that Ce6 in the form of micelles with PDT demonstrated high-contrast vascular imaging and effective vascular damage assessment [67]. Our result showed that vascular intensity was reduced by 10.4–24.5%, while vascular distribution was constricted by 28.1–32.4% after 0–3 h of Ce6-PDT treatment. However, according to Rohrbach et al., PA imaging showed a reduction of 63% average blood vessel diameter following 10 min of 2-(1-hexyloxyethyl)-2-devinyl pyropheophorbide-a (HPPH)-PDT [55]. Debefve et. al. have also reported that verteporfin caused blood vessel thrombosis after 5–15 min of PDT in a chicken embryo [29]. It can be deduced that compared to previous approaches that use damage scales to access blood vessel damage by PDT, the WGS-PAM system offers a more accurate method. In this study, the WGS-PAM system focused on the wide-field scanning of 5 mm × 8 mm (2000 pixels × 3200 pixels) to monitor the overall vascular alterations with 5 μm lateral resolution. It took 160 s in 40 mm^2^ scanning. Even though this study did not approach a single vasculature real-time monitoring, the WGS-PAM system could be utilized to offer a video rate monitoring of 25 frames/s on 200 μm × 200 μm (40 pixels × 40 pixels) to time-dependent small vessels monitoring for the future directions [91]. It will help to clearly quantify the size of close-down vessels through monitoring the blood stasis and clot formation. In addition, the 532 nm wavelength is most commonly used using the high endogenous contrast of oxyhemoglobin and deoxyhemoglobin of the photoacoustic excitation for blood vessel imaging of PAM [49,50]. At this time, the WGS-PAM system could not distinguish between oxyhemoglobin and deoxyhemoglobin, because it used a single pulse laser. However, functional photoacoustic imaging could offer multiple contrasts such as oxyhemoglobin, deoxyhemoglobin, and oxygen saturation of hemoglobin, using multiple excitation wavelength and the variability of laser parameters [50,53,69]. The functional PAM approaches could be helpful to investigate further information such as the classification of veins and arteries and oxygen saturation of hemoglobin on the PDT effects in the future.

It has been reported that Ce6-PDT-induced cell death is both dose and time dependent, and this has been validated in several in vitro and in vivo studies. Cell death induced after a dose of irradiation is known to be dependent on reactive oxygen species (ROS) production, including endothelial cell damage. In vitro tests were also conducted in the current investigation to ascertain how Ce6-PDT affects angiogenic and cell death molecules associated during pancreatic tumor regression. We demonstrated that the higher doses of Ce6-PDT attenuated the expression of angiogenic growth factors while increasing the expression of pro-apoptotic and necrotic proteins. A lower amount of VEGF expression indicates better tumor inhibition. This result corroborates with the lower level of VEGF in the lung cancer model by Ce6/SML-MSDT nanoliposomes. VEGF is a key angiogenetic factor and the vital proangiogenic growth factor secreted in tumor cells and vascular endothelial cells [92]. The outcome of VEGF expression in breast tumor tissue following sono-photodynamic therapy and Ce6 treatment also matched the published studies. Thus, the mechanism of Ce6-PDT to regress pancreatic cancer is through the stimulation of cell death signaling pathways and tumor vasculature damage, as the in vitro study and WGS-PAS imaging revealed.

The utilization of the WGS-PAM system enhanced the results of Ce6-PDT-mediated cancer treatment by monitoring photosensitizer uptake, vascular damage, and overall tumor response assessment. The WGS-PAM system’s quick imaging, mobility, and high spatial resolution can be essential for making it a promising new tool in tumor angiogenesis research. Additionally, noninvasive assessment of the extent of tumor vasculature is important in the clinical evaluation of the tumor development and treatment.

## 4. Materials and Methods

### 4.1. Preparation of Chlorin e6-Polyvinylpyrrolidone (PVP) Complex (PHONOZEN^®^)

**Figure 9:**
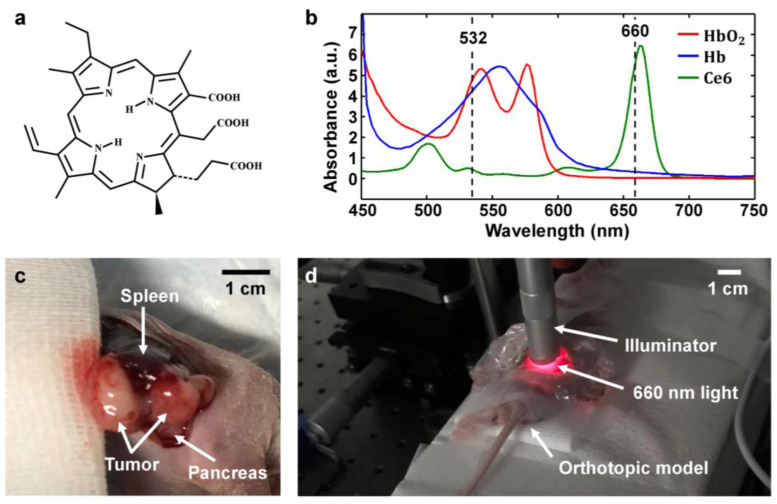
Description of photosensitizer and PDT process. (**a**) Schematic illustration of Ce6 molecular structure. (**b**) Visible spectrum of oxyhemoglobin, deoxyhemoglobin, and Ce6. (**c**) Dissected orthotopic mouse model visualizing pancreatic tumor body. (**d**) PDT setup positioning.

In the present work, Ce6 prepared in house utilizing a previously reported method, [24] has been used. The lyophilized powder for injection (Ce6-Polyvinylpyrrolidone; Ce6-PVP) in the ratio of 1:1 (PHONOZEN^®^, Dongsung, Seoul, Republic of Korea) was prepared (Figure 9a). For this, Ce6 (20 g) was suspended in water (0.8 L) and an equivalent amount of PVP preliminarily dissolved in water (0.8 L) was added to make Ce6 in the weight ratio (*w*/*w*) 1:1. An amount of 1M sodium hydroxide solution was used for maintaining a pH of about 12 to allow the formation of Ce6-trisodium salt; 1M hydrochloric acid solution was used to neutralize the solution to a pH of about 8. The final volume of 2.67 L was maintained with water to make 7.5 mg/mL solution by Ce6. Finally, 4 mL of the solution was loaded into an amber vial (30 mg Ce6/vial) and lyophilized. Thus, prepared Phonozen was characterized by UV–vis, FT-IR, HPLC, and LC-MS analysis (Figures S1–S3).

### 4.2. Cell Culture

Human pancreatic carcinoma (MIA PaCa-2) and B16F10, a murine melanoma cancer cell line obtained from the Korean Cell Line Bank (KCLB, Seoul, Republic of Korea), while human pancreas adenocarcinoma (BxPC-3-luc) cells obtained from American Type Culture Collection (ATCC) were maintained in Dulbecco’s modified Eagle medium (DMEM) containing 10% fetal bovine serum, 100 U/mL penicillin, and 100 mg/mL streptomycin. All cells were cultured at 37 °C in a humidified atmosphere of 95% air and 5% CO_2_.

### 4.3. Western Blot

MIA PaCa-2 and BxPC-3 cells were respectively seeded in six-well plates at a density of 2.5 × 10^5^ cells/well and treated with different concentrations of Ce6 (0.1 and 2 μΜ). After 3 h of incubation, cells were treated with the LED light (660 nm, 50 mW, 5 J/cm^2^) and incubated for an additional 24 h. To isolate total protein from the respective cells, cells were homogenized with RIPA lysis buffer (150 mM Sodium chloride, 1% Triton X-100, 0.5% Sodium deoxycholate, 0.1% SDS and 50 mM Tris adjusted to pH 8.0) containing 1× proteases and phosphatases inhibitors for 1 h. The lysates were centrifuged at 10,000× *g* for 10 min at 4 °C to remove cell debris. Proteins were quantitated and were separated using 10% SDS–polyacrylamide gel electrophoresis (SDS-PAGE) and electrotransferred in Immobilon P membranes (Millipore Corp., Bedford, MA, USA). The membranes were blotted with different primary antibodies diluted in blocking solution at 4 °C: anti-rabbit anti-bax (bs-0127R, Bioss, Woburn, MA, USA), anti-mouse anti-Bcl-2 (bs-52022M, Bioss, Woburn, MA, USA), anti-rabbit β-Actin (8226, Abcam, Cambridge, UK) antibodies, anti-rabbit anti-PARP-1 (CST9532), anti-rabbit caspase-3 (CST9662), anti-rabbit anti-survivin (CST2808), anti-rabbit anti-p-MLKL(CST91689), anti-rabbit anti-MLKL(CST14993S) and VEGF (ab46154). The protein band was visualized with an enhanced chemiluminescence (ECL) reagent and the blot images were obtained by a luminescent image analyzer (Amersham, GE Healthcare, Chicago, IL, USA).

### 4.4. Preparation of Orthotopic Pancreatic Cancer Mouse Model for In Vivo Imaging

All the animal experiments were performed in accordance with the guidelines of the Institutional Animal and Human Care and Use Committee of Kyungpook National University (No. KNU-2018-0100). Balb/c nu/nu mice (*n* = 30) were purchased from Orient Bio Inc. (Seongnam, Republic of Korea). At six weeks of age, mice underwent anesthesia, followed by a left subcostal incision. Pancreatic cancer cells (BxPC-3-luc, 5 × 10^5^) were mixed with a 1:1 ratio of media and Matrigel (20 µL). These cancer cells were then implanted into the pancreas beneath the spleen of the mice using a 30-gauge insulin needle. There was no appearance of a fluid bleb and intra-peritoneal leakage when tumor cell implantation was completed. The wound was sutured aseptically after the procedure. Under general anesthesia, routine preparation and drapes were performed. The anesthesia was performed using 0.75% isoflurane (Oxygen: 1 L/min) and confirmed by monitoring the movements of hands and feet. During the experiment, the animal body was placed on a heating pad to maintain the body temperature. The illumination beam on the target region of the mouse model followed the American National Standard Institute safety limit of 532 nm.

### 4.5. Injection of Photosensitizer and Irradiation by Light-Emitting Diodes for Activation

The mice were given 2.5 mg/kg Ce6 solution through bolus tail vein injection. The Ce6 was prepared by dissolving in normal saline as a vehicle for intravenous drug administration. Mice were incubated for 3 h as per the protocol suggested by our previous paper [23,24], and tumors were irradiated with a laser of 660 nm. The absorption spectrum of oxyhemoglobin, deoxyhemoglobin, and Ce6 is plotted in Figure 9b. All three oxyhemoglobin, deoxyhemoglobin, and Ce6’s displayed a higher absorption band at around 532 nm. On the other hand, Ce6 absorption was significantly higher (18 fold) than that of oxyhemoglobin and deoxyhemoglobin at 660 nm. Therefore, the light of the 660 nm wavelength was selected for Ce6 irradiation. The pancreatic tumor body along with the pancreas and spleen in the orthotopic mouse model is shown in Figure 9c. The flattest surface of the tumor tissue was selected for scanning. After dissection, the blood was removed, and the scanning region was washed with phosphate-buffered saline. The process of tumor tissue irradiation at 660 nm is presented in Figure 9d. The diameter of the collimated beam illuminating on the tumor tissue was 11.3 mm. PDT was performed using the light-emitting diode after intravascular injection of the photosensitizer under the dark condition. A beam of 660 nm having power of 200 mW/cm^2^ (200 J, for 1000 s) was illuminated at the imaging region passing through the ultrasonic gel to activate the PDT effect.

### 4.6. Waterproof Galvanometer Scanner Photoacoustic Microscopy

**Figure 10:**
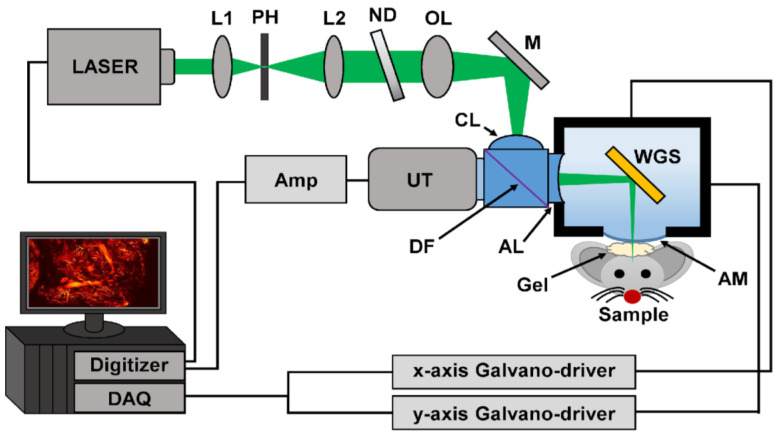
Schematic representation of the waterproof galvanometer scanner photoacoustic microscopy system. L: lens, PH: pinhole, ND: neutral density filter, OL: objective lens, M: mirror, Amp: amplifier, UT: ultrasound transducer, CL: correction lens, DF: dielectric film, AL: acoustic lens, AM: acoustic membrane, WGS: waterproof galvanometer scanner, DAQ: data acquisition board.

The developed WGS-PAM system configuration is illustrated in Figure 10. A Q-switch-diode-pumped-solid-state laser (SPOT-10-200-532, Elforlight Ltd., Daventry, UK) was used with a wavelength of 532 nm. In WGS-PAM operation, a 40 kHz repetition rate was used with an external triggering to synchronize the axial amplitude scan rate and the signals of a digitizer. An objective lens with a 75 mm focal length and an acoustic lens with a 27 mm focal length were used. The dielectric film in the beam combiner facilitated the reflection of both the optical beam and transmission of the acoustic wave. The ultrasonic transducer had a 50 MHz center frequency with an approximate 100 MHz full width at half maximum. Further technical information along with functional parameters of the advanced WGS-PAM system can be found elsewhere [91,93]. The WGS-PAM offers the 4.9 µm of lateral resolution and 9 mm by 14.5 mm of the maximum x–y plane scan-region.

### 4.7. A Timeline of the WGS-PAM Imaging with PDT

A timeline of the experiments describes the comprehensive timing of WGS-PAM imaging and PDT treatment for better understanding. First, control imaging was conducted using the WGS-PAM system after dissection and positioning on the benchtop table. Second, the circulation of Ce6 waited for 1 h after the intravascular injection of the photosensitizer under the darkroom condition. Third, the irradiation was performed using the light-emitting diode to activate the PDT effect for 1000 s. Lastly, the WGS-PAM system monitored the PDT-treated tumor area every 30 min for 3 h, as shown in Figure 6. The shams were also monitored to compare the PDT effects using the WGS-PAM system with the same time intervals.

### 4.8. Visualization of Shams and Photodynamic Therapy Treatments

The shams indicate three groups, namely, those of only anesthesia, injection of the photosensitizer without the irradiation, and irradiation without the injection of the photosensitizer to clearly compare the PDT effects. The shams show each effect of anesthesia, photosensitizer, and irradiation with the WGS-PAM imaging. The shams were categorized using three different methods: anesthesia (*n* = 3), injection of photosensitizer (*n* = 1), and irradiation (*n* = 1). All shams were visualized using the WGS-PAM system at 30 min intervals for 3 h in each condition. PDT-treated cases (*n* = 5) were visualized in the same manner. First, the tumor models were imaged using the WGS-PAM system before injecting the photosensitizer for the control images. Second, the models were monitored after injecting the photosensitizer. Lastly, the models were monitored after activating PDT treatment at 30 min intervals for 3 h. The field of view of the PAM images is 5 mm × 8 mm (2000 pixels × 3200 pixels) with 20 frames/s of 5 mm B-scan. It took 160 s for each scanning of 40 ${mm}^{2}$. The brightness of the PAM absorption intensity is arranged to 0–255 scales.

### 4.9. Epi-Fluorescence and Luminescence Analysis

One important aspect of PDT is vascular stasis, which can be observed with fluorescence imaging. With the aid of a near-infrared-labeled fluorescent dye AngioSense 750 EX (PerkinElmer, Waltham, MA, USA), tumor vascularization was detected. The dye is known to preferentially bind to newly developed blood vessels. The anesthetized mice (*n* = 3) with the pancreatic tumor of BxPC-3-luc cells before and after 24 h of PDT underwent fluorescence imaging following the intravenous injection of dye through the tail vein (excitation: 745 nm; emission: 800 nm; high pass filter cut off: 770 nm; illumination). The angiogenic process is monitored using an in vivo imaging system (IVIS). Retained fluorophores were most abundant in the tumor regions 24 h after injection.

Anesthetized mice (*n* = 2) with the pancreatic tumor of BxPC-3-luc cells before and after 24 h of PDT were administered with the intraperitoneal injection of D-Luciferin dissolved in sterile PBS. Each image was saved for subsequent analysis. Images were acquired every 2 min for 30 min (10 s exposure/image). Signal intensity from an equal-sized ROI of the tumors from tumor-bearing animals in the control was quantitatively assessed using Living Image 4.4 software (IVIS Imaging Spectrum System, Caliper, Newton, MA, USA).

### 4.10. Histopathological Analysis

All experimental animals (*n* = 5 per treatment group) were euthanized at the end of the study at which time tumors were harvested and fixed with 10% buffered formalin, embedded in paraffin and sectioned. Sections were cut at 5 µm thickness using an automatic microtome. All samples were stained with hematoxylin and eosin (H&E) and sections were evaluated for microvessels using a light microscope. All 3 tumors from each treatment group were evaluated for histology analysis. After scanning the section and storing the images, the highest density of blood vessels was determined under 100× magnification of the original image, and then under 200× magnification.

## 5. Conclusions

This research has qualitatively and quantitatively assessed the vascular shutdown effect of Ce6-associated PDT using the high-speed wide-field WGS-PAM system on in vivo label-free tissues of an orthotopic pancreatic cancer mouse model. The micro blood vessels of a pancreatic tumor were monitored using the developed WGS-PAM system after conducting the PDT treatment at 30 min intervals for 3 h. The variation of microvasculature was visualized by the absorption intensity of PAM and the structural distribution of vessels. The averaged vascular intensity decreased by 15.4% and 24.5% at 0 h and 3 h post-PDT, respectively. The average vascular distribution was constricted by 28.1% and 32.4% after 0 h and 3 h of PDT, respectively. To the best of our knowledge, this is the first demonstration of the effects of PDT on in vivo orthotopic pancreatic cancer tissue by the developed WGS-PAM system. The high-speed wide-field WGS-PAM system is anticipated to be effectively utilized to investigate the angiographic dynamics of the tumor tissues and treatment progress in high-contrast label-free in vivo microvessel imaging for the orthotopic pancreatic cancer model. The resolution (5 μm) was adequate for the detection of microvascular damage; however, the system’s lateral resolution can still be increased.

## Data Availability

The data presented in this study are available on request from the corresponding author. The data are not publicly available due to privacy and confidential restriction.

## Supplementary Materials

The following supporting information can be downloaded at: https://www.mdpi.com/article/10.3390/ijms25063457/s1.
