# Supplementary material for: Efficient Assessment of Tumor Vascular Shutdown by Photodynamic Therapy on Orthotopic Pancreatic Cancer Using High-Speed Wide-Field Waterproof Galvanometer Scanner Photoacoustic Microscopy
Source: Int J Mol Sci. 2024 Mar 19;25(6):3457. doi: 10.3390/ijms25063457 (PMC10970422; doi:10.3390/ijms25063457)
Supplement: Supplementary file 1 [file ijms-25-03457-s001.zip › ijms-2861873-supplementary.pdf]

Supplementary Materials

Efficient Assessment of Tumor Vascular Shutdown by Photodynamic Therapy on Orthotopic Pancreatic Cancer Using High-Speed Wide-Field Waterproof Galvanometer Scanner Photoacoustic Microscopy

Jaeyul Lee [1,2,†], Sangyeob Han [1,3], Til Bahadur Thapa Magar [4], Pallavi Gurung [4], Junsoo Lee [1], Daewoon Seong [1], Sungjo Park [5], Yong-Wan Kim [4,*], Mansik Jeon [1,*] and Jeehyun Kim [1]

[1] School of Electronic and Electrical Engineering, College of IT Engineering, Kyungpook National University, Daegu 41566, Republic of Korea
[2] Organic Nanoelectronics Laboratory, KNU Institute for Nanophotonics Applications (KINPA), Department of Chemical Engineering, School of Applied Chemical Engineering, Kyungpook National University, Daegu 41566, Republic of Korea
[3] Institute of Biomedical Engineering Research, Kyungpook National University, Daegu 41566, Republic of Korea
[4] Dongsung Cancer Center, Dongsung Bio Pharmaceutical Co., Ltd., Daegu 41061, Republic of Korea
[5] Laser Application Center, Institute of Advanced Convergence Technology, Kyungpook National University, Daegu 41061, Republic of Korea
* Correspondence: kyw17@ds-pharm.co.kr (Y.-W.K.); msjeon@knu.ac.kr (M.J.)
† Current address: Division of Pulmonary and Critical Care Medicine, Massachusetts General Hospital, Harvard Medical School, Boston, MA 02114, USA.

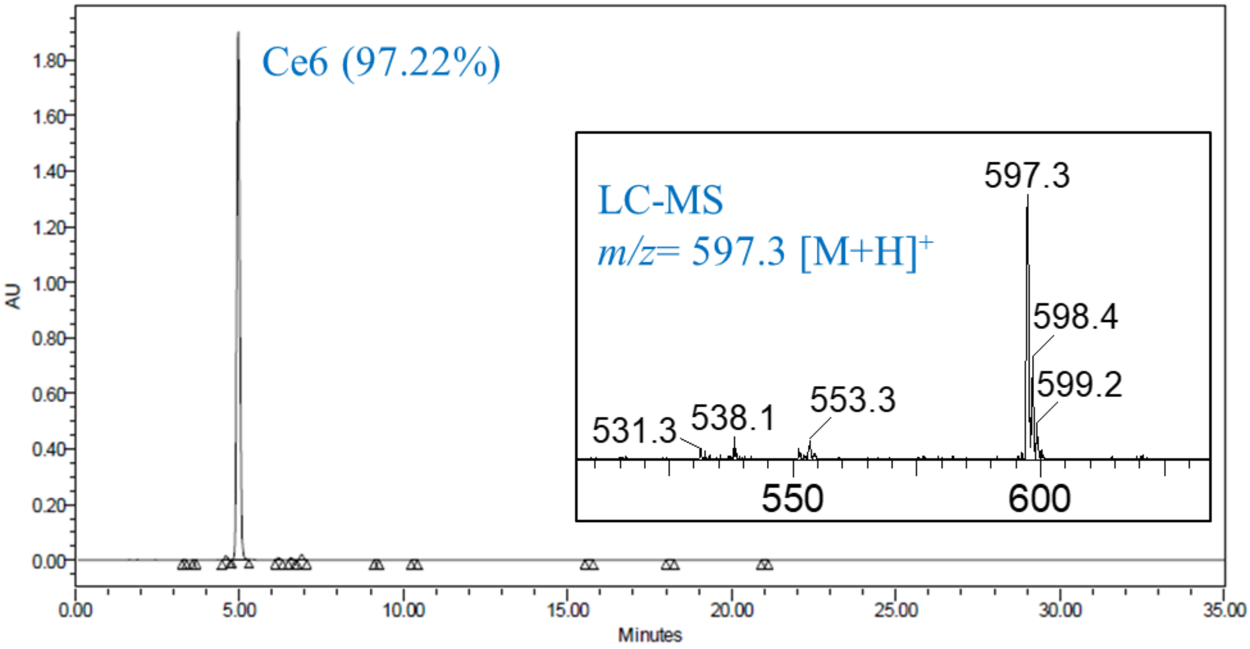

Figure S1. HPLC & ESI-MS chromatogram of Ce6.

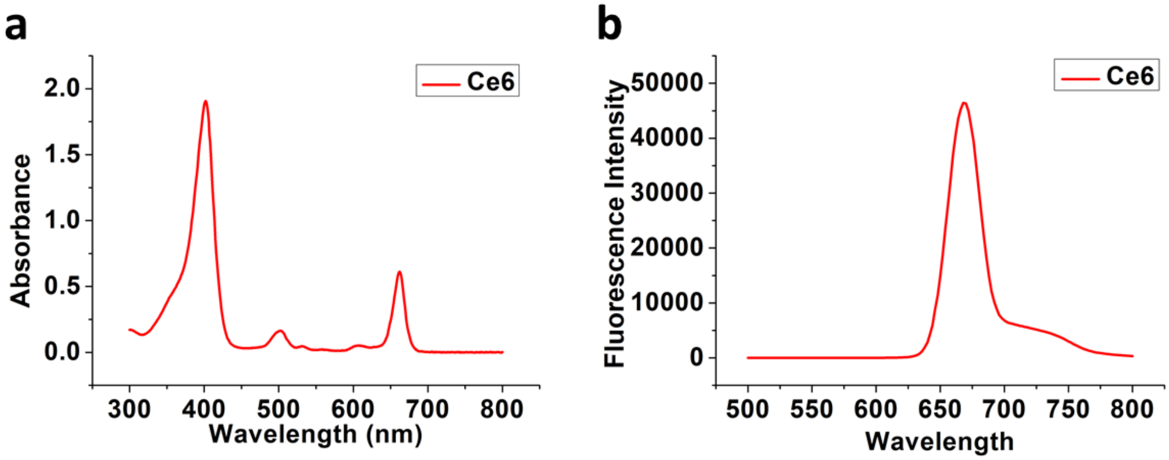

Figure S2. UV-visible (a) and fluorescence spectra (b) of Ce6.

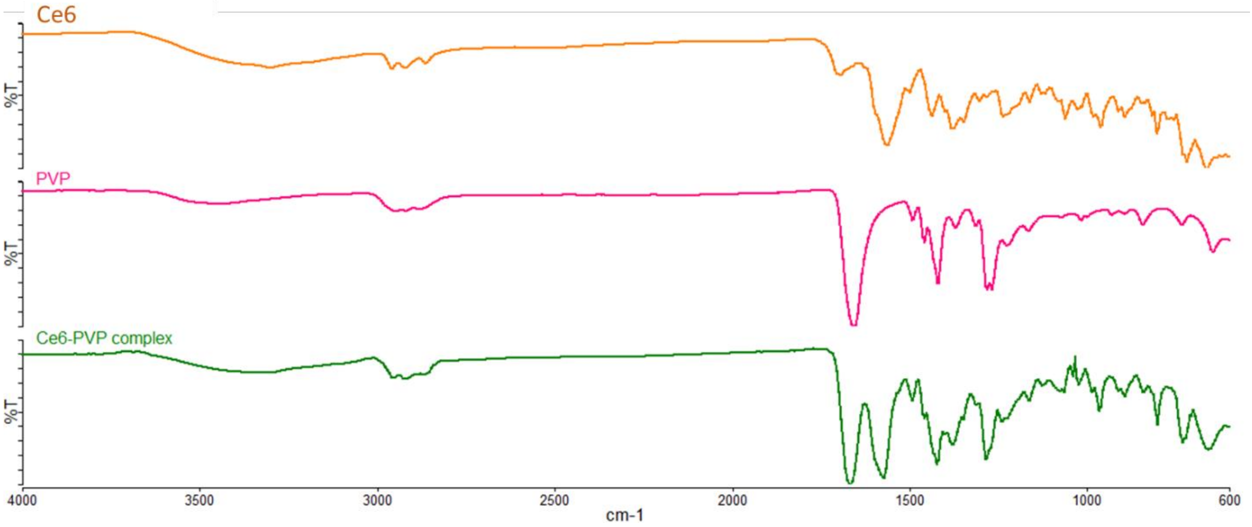

Figure S3. FT-IR fingerprint of Ce6, PVP, and Phonozen (Ce6+PVP).

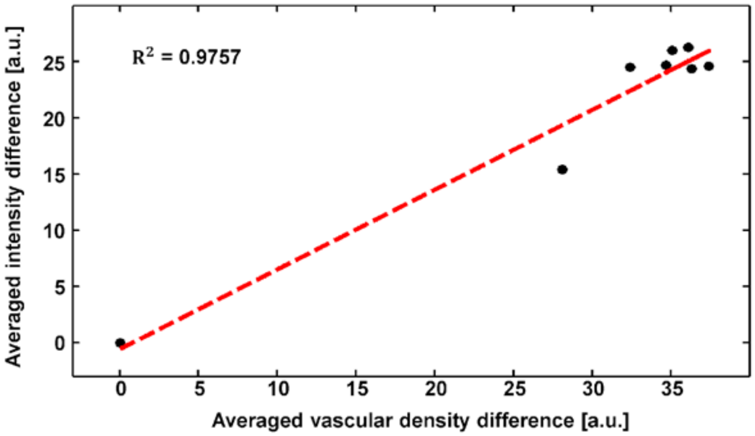

Figure S4. Correlation between the intensity and the vascular density difference of shams and post-PDT.

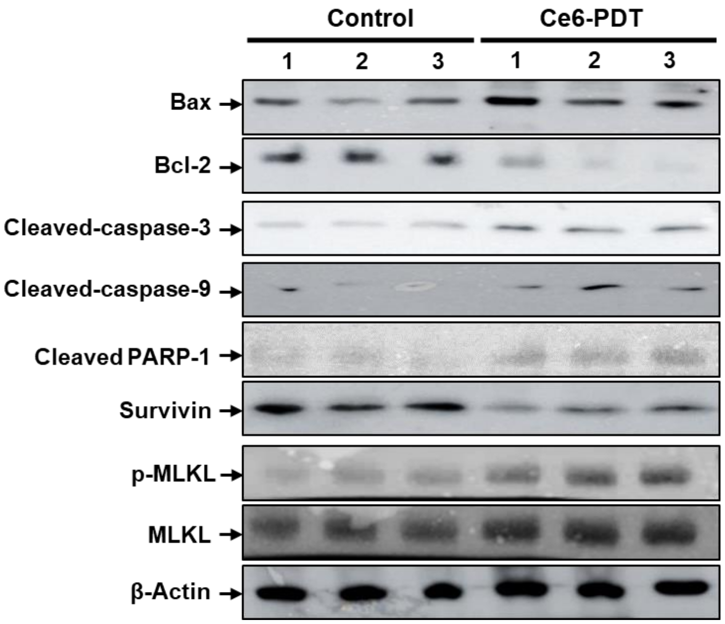

Figure S5. Levels of apoptotic and necroptotic marker proteins in mouse melanoma tumors suggest a protective action of Ce6-PDT. Mouse were intravenously injected with Ce6 (2.5 mg/kg) in a xenograft mouse model (B16F10 cells) for 3 h followed by irradiation with 660 nm laser, 100 J/cm^2 for 8 min 20 s. β-actin was used as a loading control.

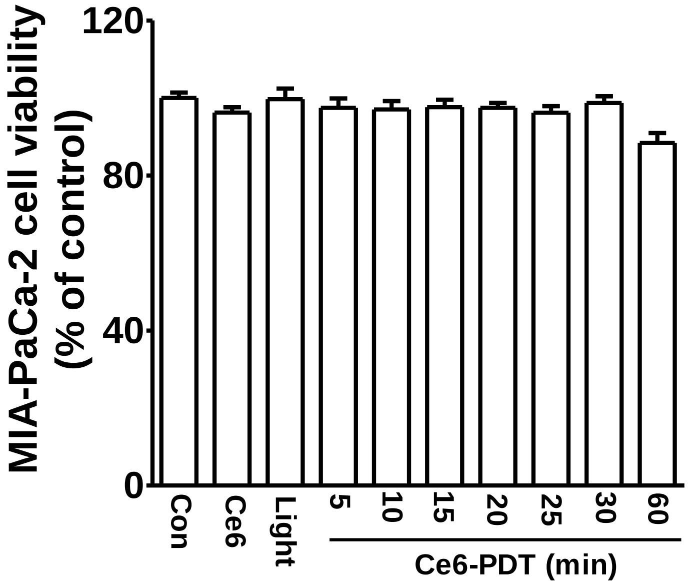

Figure S6. Cell Viability analysis by MTT assay in MIA-PaCa-2 cell lines treated with Ce6-PDT. Cells were first incubated with 20 μM Ce6 in medium for 3 h and then they were irradiated with a light dose of 0.5 J/cm^2 using a LED light source (620 nm). MTT assays were performed in 5, 10, 15, 20, 25, 30 and 60 min after irradiation. (Data are the representative of three replicates)

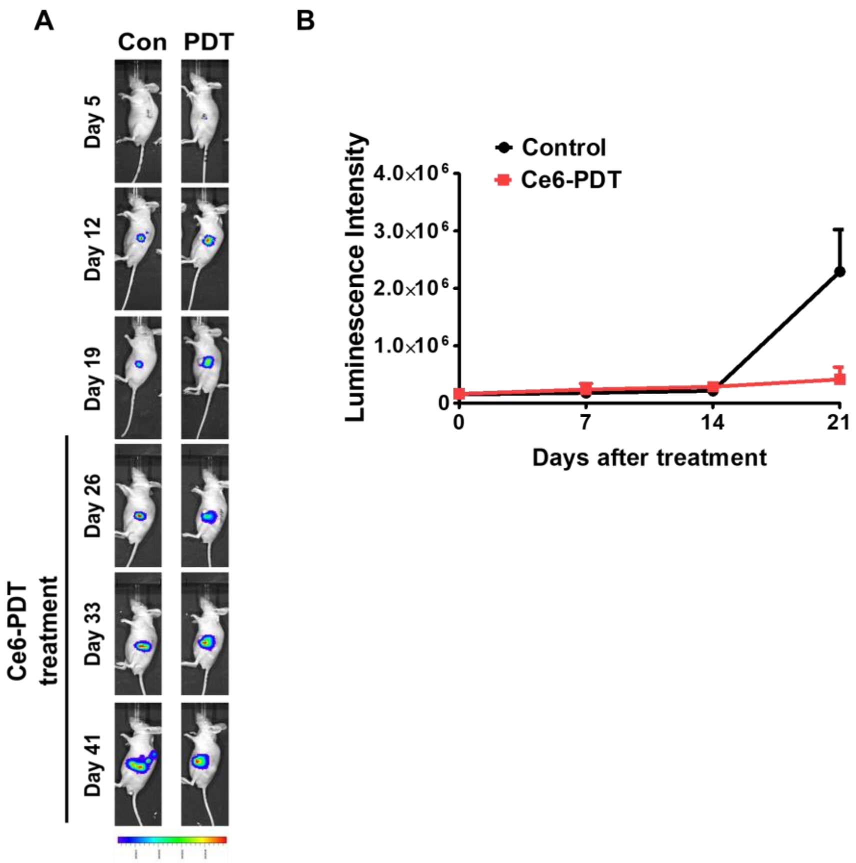

Figure S7. Amelioration of orthotopic pancreatic cancer by Ce6-PDT. Orthotopic pancreatic cancer mouse models (nude mice) were developed by intrapancreatic injection of BXPC-3- luc cell line. At 18 days after tumor injection, mice were treated with intravenous injection of 2.5 mg/kg of Ce6 followed by laser irradiation of 100 J/cm^2 for 8 min 20s. Development of tumors were then tracked by IVIS imaging (n=5 each group). A. Variations in the IVIS-monitored signal produced in tumors from representative mice in control and Ce6-PDT groups. B. Luminesce intensity of photons emitted by each tumors are displayed in the graph.
